# High Doses of Ursodeoxycholic Acid Up-Regulate the Expression of Placental Breast Cancer Resistance Protein in Patients Affected by Intrahepatic Cholestasis of Pregnancy

**DOI:** 10.1371/journal.pone.0064101

**Published:** 2013-05-24

**Authors:** Francesco Azzaroli, Maria Elena Raspanti, Patrizia Simoni, Marco Montagnani, Andrea Lisotti, Paolo Cecinato, Rosario Arena, Giuliana Simonazzi, Antonio Farina, Nicola Rizzo, Giuseppe Mazzella

**Affiliations:** 1 Department of Medical and Surgical Sciences, S.Orsola-Malpighi Hospital, University of Bologna, Italy; 2 Centre for Applied Biomedical Research, S.Orsola-Malpighi Hospital, Bologna, Italy; Centro di Riferimento Oncologico, IRCCS National Cancer Institute, Italy

## Abstract

**Background:**

Ursodeoxycholic acid (UDCA) administration in intrahepatic cholestasis of pregnancy (ICP) induces bile acids (BA) efflux from the foetal compartment, but the molecular basis of this transplacental transport is only partially defined.

**Aim:**

To determine if placental breast cancer resistance protein (BCRP), able to transport BA, is regulated by UDCA in ICP.

**Methods:**

32 pregnant women with ICP (14 untreated, 34.9±5.17 years; 18 treated with UDCA - 25 mg/Kg/day, 32.7±4.62 years,) and 12 healthy controls (33.4±3.32 years) agreed to participate in the study. Placentas were obtained at delivery and processed for membrane extraction. BCRP protein expression was evaluated by immunoblotting techniques and chemiluminescence quantified with a luminograph measuring emitted photons; mRNA expression with real time PCR. Statistical differences between groups were evaluated by ANOVA with Dunn’s Multiple Comparison test.

**Results:**

BCRP was expressed only on the apical membrane of the syncytiotrophoblast. A significant difference was observed among the three groups both for mRNA (ANOVA, p = 0.0074) and protein (ANOVA, p<0.0001) expression. BCRP expression was similar in controls and in the untreated ICP group. UDCA induced a significant increase in placental BCRP mRNA and protein expression compared to controls (350.7±106.3 vs 100±18.68% of controls, p<0.05 and 397.8±56.02 vs 100±11.44% of controls, p<0.001, respectively) and untreated ICP (90.29±17.59% of controls, p<0.05 and 155.0±13.87%, p<0.01).

**Conclusion:**

Our results confirm that BCRP is expressed only on the apical membrane of the syncytiotrophoblast and show that ICP treatment with high dose UDCA significantly upregulates placental BCRP expression favouring BA efflux from the foetal compartment.

## Introduction

Intrahepatic cholestasis of pregnancy (ICP), a liver disorder unique to pregnancy, predominantly occurs during the third trimester of gestation. ICP is characterized by pruritus and biochemical alterations in liver tests which fully resolve after delivery [1], [2], and is associated with an increased risk of foetal distress, preterm delivery and sudden intrauterine foetal death [3]. Given the substantial foetal risk, ICP is widely considered a serious clinical problem [3].

Various hypothesis involving environmental, genetic and hormonal factors have been investigated to elucidate the pathogenesis of ICP. While no certain cause has been identified, impaired bile acids (BA) metabolism leads to increased BA accumulation in the foetal compartment [4]. Elevated maternal BA levels affect placental transport, placental hormone production and chorionic vessel constriction [5]–[8] and increase myometrial sensitivity to oxytocin [7], [9].

The placenta plays a major role in the pathophysiology of adverse foetal effects because of its capacity to transport BA. This capacity may somehow be regulated by ursodeoxycholic acid (UDCA), the treatment of choice for ICP [3], [10]. Previous studies have shown that UDCA may improve bile acid transport across the placenta and upregulate the expression of transporters localized on the apical side of the trophoblast membrane. However, the classical transporters studied to date are not good candidates for primary bile acid. The breast cancer resistance protein (BCRP) has been reported to transport BA [11] but its placental expression in ICP has not been evaluated. Therefore, our study aimed to determine BCRP expression in ICP and its regulation by UDCA administration.

## Materials and Methods

### Ethics Statement

All women gave verbal informed consent to the study that was carried out according to the Declaration of Helsinki. All subjects were older than 18 years and able to give informed consent. The study was limited to the placenta which is routinely discarded as waste material and there was no change to the current standard medical and obstetric management of the patients. In addition, the study involved no sensitive genetic data or investigations with clinical consequences on the newborn or the mother. For these reasons, ethics approval was not required according to the Italian legislation (Law n.211, 24 June 2003, Directive 2001/20/CE). However, a letter from the gastroenterologist to the gynaecologist/obstetrician reporting patients’ verbal consent was attached to the clinical records.

### Patients

Thirty-two consecutive pregnant women with ICP were enrolled in the study. A control group comprised 12 consecutive healthy women with physiological pregnancies. The following criteria were required for the diagnosis of ICP: elevated fasting serum BAs (>10 µmol/L), elevated aminotransferases (>40 IU/L for ALT and >37 IU/L for AST), and pruritus. Exclusion criteria were infectious, metabolic or drug-related liver disease, and a history of alcohol or drug abuse. Blood tests and abdominal ultrasonography were performed to exclude obstructive cholestasis and viral [anti-HCV antibodies [III generation enzyme-linked immunosorbent assay (ELISA)], HBsAg, anti-Epstein–Barr virus, anti-CMV, anti-HSV, anti-HIV], metabolic (blood cholesterol, glucose, triglycerides and iron) or autoimmune (ANA, SMA, AMA, p-ANCA and LKM) liver diseases.

Information on available treatments and their implications in ICP was provided by the same doctor (GM) to all patients on enrolment. Despite literature data suggesting that UDCA is the first choice treatment in ICP, this indication is off-label in Italy. Consequently, as the present study was not designed to evaluate drug efficacy, patients were free to choose which arm to belong to (treatment = group1/no treatment = group2). Therefore, treated patients constituted group 1 while women with ICP who refused UDCA treatment were included in the untreated group 2. Group 1 consisted of 18 ICP patients who received UDCA 25 mg/kg/day; Group 2 consisted of 14 ICP patients who received only diet and bed rest. Antihistaminics were allowed in case of intolerable pruritus (leading to insomnia/restlessness).

### Placenta Collection and Membrane Vesicle Preparation

Placentas were obtained from term delivery or caesarean section (38–41 weeks) together with samples of cord and maternal blood. All placentas were immediately washed with cold PBS, placed on ice and processed for membrane separation or frozen in liquid nitrogen. Apical membranes were prepared according to Grube et al. [12] and the degree of enrichment in trophoblastic vesicles was assessed by determination of alkaline phosphatase and ATP-ase activity [13]. Briefly, pieces of placenta were homogenized (UltraTurrax) for 2 min in lysis buffer (250 mM sucrose, 10 mM Tris-HEPES pH 7, 4 mM EGTA, 5 mM EDTA) and centrifuged at 9000 *g* for 10 min; the supernatant was centrifuged at 100000 g for 35 min. The resulting pellet was resuspended in the lysis buffer containing MgCl_2_ (final concentration 10 mM), stirred on ice for 10 min and then centrifuged at 2500 *g* for 15 min. The supernatant, corresponding to the apical fraction and the pellet, containing basolateral membranes were rehomogenized (Potter), centrifuged at 100000 g per 35 min and the resulting pellets were resuspended in 20–80 µl of buffer. The membrane protein fractions were stored at –80°C until used.

### Western Blot

Protein concentrations were determined according to the method of Lowry et al. [14] and equal amounts of membrane proteins (100 µg) were separated by standard SDS-PAGE electrophoresis. Actin was used as an internal control (AC-40, Sigma-Aldrich, St Louis, MO, USA), giving a constant signal in all samples. After transfer onto nitrocellulose membrane, immunoblotting was performed with an overnight incubation with anti-BCRP antibodies [anti ABCG2 (BXP21)sc-58222, anti ABCG2 (H70)sc-25821dil 1∶100 in TBS-Tween, Santa Cruz Biotechnology, Santa Cruz, CA, USA]. After a 1 h incubation at room temperature with the peroxidase-conjugated secondary antibodies (Envision antimouse IgG; K4001 and Envision antirabbit, K4003, Dako, Glostrup, Denmark), chemiluminescent signals (ECL, Amersham Pharmacia Biotech, Milan, Italy) were revealed by a Luminometer (Molecular Light Imager, Berthold Technologies, Bad Wildbad, Germany). The chemiluminescent signal was quantified by Winlight 32 analysis software (Version 2.91, Berthold Technologies).

### Real-time PCR

Total RNA was extracted from 50–100 mg frozen placental tissue using TrizolReagent (Invitrogen, Milan, Italy) according to the manufacturer’s instructions. Purity of extracted RNA was measured by the adsorbance ratio 260/280 nm (1.8–2.0) and its integrity was controlled on 1% agarose gel. Two µg of RNA were converted to first-strand cDNAs with the Superscript III reverse transcriptase kit (Invitrogen, Life Technologies Europe BV, Monza, Italy) according to the manufacturer’s instructions.

Real-time PCR was performed with the following primer and probe sets (TaqMan Gene Expression Assays) diluted 1∶20, purchased from Applied Biosystems Italia (Monza, Italy):B-Actin (Human beta actin, cat. N. 4333762F), BCRP (Hs01053786_m1). Two µl of cDNA were added to TaqMan Universal Master Mix (Applied Biosystems) and amplified in duplicate in an Applied Biosystems 7000 real-time PCR cycler. Cycle conditions were 50°C for 2 min and 95°C for 10 min, then cycled to 95°C for 15 s and 60°C for 1 min for 40 cycles. All samples were normalized to B-actin expression levels and the relative quantification was performed using the Ct method.

### Statistics

Statistical differences between groups were evaluated by ANOVA with Dunn’s Multiple Comparison test and the results were expressed as mean ± S.E. Chi-squared test and Mann-Whitney test were used when appropriate.

## Results

The groups were comparable with regard to age, parity and type of delivery. As expected a significant difference was present between controls and the cholestatic groups with regard to week of delivery, serum bile acids and transaminase levels. No significant differences were observed between the treated and untreated cholestatic groups (table 1).

**Table 1:** Baseline characteristics of the studied groups.

	CONTROLS	ICP	ICP+UDCA	p≤
	(N = 12)	(N = 14)	(N = 18)	
***Age***	33.4±3.32	34.9±5.17	32.7±4.62	ns
***Week of delivery***	39.17±1.4*	36.64±1.80	35.50±2.59	0.0003
***Transaminases***				
* GOT*	23.0±6.54*	139.1±74.81	135.4±146.2	0.0001
* GPT*	16.60±5.98*	233.2±45.85	217.0±238.6	0.0001
***Bile acids***				
* Cholic Acid*	0.62±0.76*	16.80±17.52	20.53±14.46	0.0002
* Chenodeoxycholic Acid*	0.63±0.66*	8.15±8.55	12.71±8.50	0.0002
***Type of delivery***				
* Vaginal*	9	9	8	ns
* Caesarean*	3	5	10	ns
***Parity***				
* Primiparae*	9	12	14	ns
* Multiparae*	3	2	4	ns

* vs ICP and ICP+UDCA.

**Figure 1:**
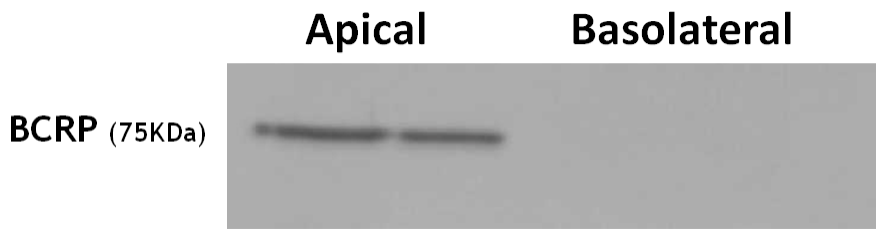
Western blotting of syncytiotrophoblast membranes showing BCRP expression only on their apical side.

**Figure 2:**
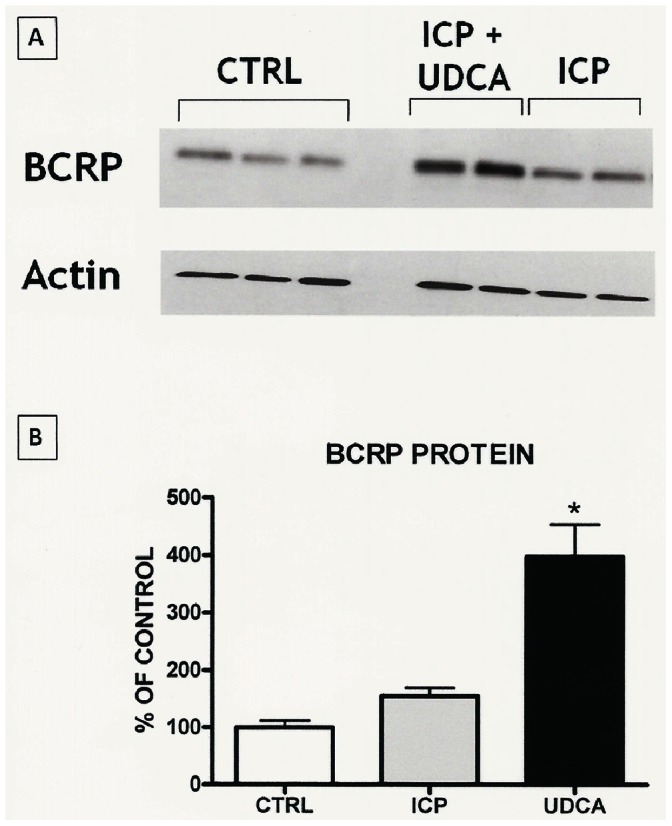
Results of immunoblotting quantification showing BCRP protein expression. A: an example of western blotting of BCRP protein expression on the apical membranes of the syncytiotrophoblast in the different groups under study. B: results of BCRP protein quantification. *ANOVA – P<0.0001 (Dunn’s Test: ICP+UDCA vs CTRL, P<0.001; ICP+UDCA vs ICP, P<0.01).

**Figure 3:**
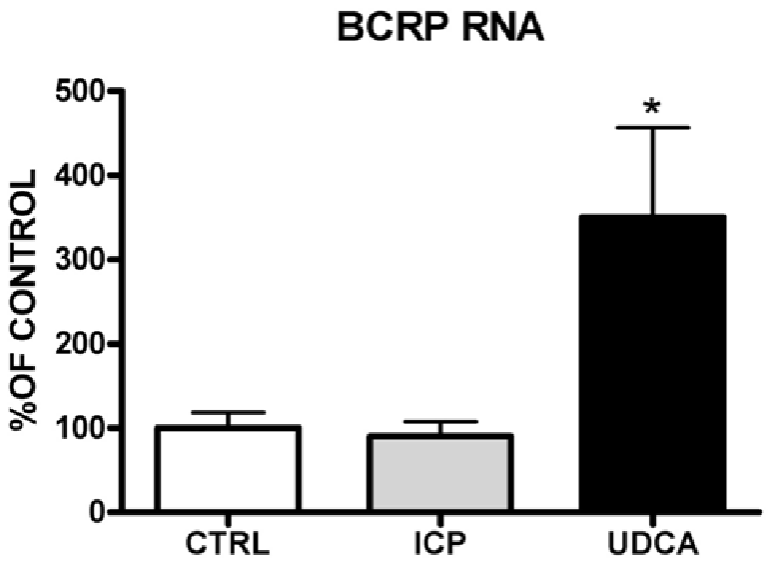
Results of RT-PCR quantification showing BCRP mRNA expression on the apical membranes of the syncytiotrophoblast in the different groups under study. *ANOVA – P = 0.0074 (Dunn’s Test: ICP+UDCA vs CTRL and vs ICP, P<0.05).

Immunoblotting of apical and basolateral membranes confirmed that BCRP is expressed only on apical membranes of the syncytiotrophoblast (figure 1). Then the evaluation of apical membranes isolated from placentas collected from controls and groups 1 and 2 showed that the BCRP protein was significantly (p<0.0001) increased in group 2 (397.8±56.02%) compared to both group 1 and controls (155.0±13.87% and 100.0±11.44%, respectively) (figure 2). Similarly, BCRP mRNA expression was significantly (p = 0.0074) increased in group 2 (350.7±106.3%) vs group 1 and controls (90.29±17.59% and 100.0±18.68%, respectively) (figure 3).

The type of delivery (vaginal or caesarean section) did not significantly influence placental BCRP mRNA or protein levels within the groups (see table 2).

**Table 2:** BCRP data according to type of delivery.

	CTRL	ICP	UDCA
	Vaginal #9	Vaginal #9	Vaginal #8
	Caesarean #3	Caesarean #5	Caesarean #10
***Protein (% of control)***			
* Vaginal*	97.14±15.03	161.5±18.49	411.9±74.62
* Caesarean*	108.5±10.85	141.9±20.86	376.6±91.95
***P***	ns	ns	ns
***mRNA (% of control)***			
* Vaginal*	106.0±24.33	87.30±20.25	361.2±201.5
* Caesarean*	86.11±30.87	98.29±42.42	341.6±109.0
***P***	ns	ns	ns

## Discussion

Our study shows that BCRP protein is upregulated in placentas from ICP women receiving UDCA compared to untreated patients and controls. We observed no significant difference in BCRP levels between samples from vaginal or caesarean section within each group suggesting that labour does not affect BCRP expression. This observation is more consistent for group 1 because of comparable numbers of caesarean and vaginal deliveries.

UDCA has been shown to improve bile acids (BA) transport across the placenta both via ATP-dependent and independent mechanisms [15]. The molecular basis of this transport remains unsettled. In humans, the only ATP-dependent molecule found modestly upregulated by UDCA in ICP is multidrug resistance protein 2 (MRP2) [16]. Furthermore, MRP2 predominantly transports bilirubin and to a minor extent sulphated BA [17], [18], suggesting that other proteins are involved in BA transplacental transport.

A few years ago, studies on BCRP-expressing bacteria showed that BCRP can transport primary BA [11], but subsequent studies in cells transfected with BCRP failed to show transport for taurocholic acid and or taurolithocholate sulphate [19]–[21]. More recently, in vitro and in vivo evidence in knockout mice suggests that BCRP is able to transport BA across the epithelia that express it including the placenta [22]. Recent findings in knockout mice suggest that, despite a minimal/no role in the liver’s adaptive response to cholestasis, BCRP might be implicated in solute export in the kidney and intestine suggesting that its role in cholestasis might be more prominent in extrahepatic tissues [23]. In this regard, the placenta is a unique extrahepatic tissue that loses expression of the main BA transporters (BSEP and NTCP) during the course of gestation. Therefore, in the third trimester placenta, specific BA transport is likely to be covered in part by other transporters sharing a similar substrate specificity. ICP is typical of the third trimester when oestrogen levels peak and favour cholestasis while the putative BA transporters (BSEP and NTCP) are not expressed in the human placenta despite the need for BA transport.

In this setting, the presence of BCRP on the apical membrane of the syncytiotrophoblast may suggest that the protein could contribute to BA transport across the human placenta towards maternal blood. We do not know whether the BCRP contribution is tangible or not in humans, but literature evidence suggests that this might be the case in mice [24].

Surprisingly, we did not observe a BCRP upregulation in untreated patients compared to controls suggesting that placental BCRP does not have an adaptive response in non obstructive cholestasis in humans. Interestingly, BCRP was upregulated in treated patients both at mRNA and protein levels, but the mechanism by which UDCA induces a transcriptional upregulation of BCRP is not straightforward. BCRP is regulated by various endogenous and xenobiotic factors including hormones (progesterone and oestrogens) [25], [26], highlighting the role of progesterone and oestrogen regulation of BCRP in pregnancy. The effect of oestradiol and progesterone has been studied in vitro with contradictory results for both. Overall, the final effect of these hormones on BCRP regulation seems to depend on their relative levels and the expression of progesterone receptors B and A as well as the ratio of the progesterone receptors (progesterone receptor A/progesterone receptor B). BCRP is regulated by a number of nuclear receptors including the peroxisome proliferator-activated response (PPARγ, hypoxia inducible factor (HIF-1), aryl hydrocarbon receptor (AhR) and pregnane X receptor (PXR).

UDCA is not considered an effective ligand for nuclear receptors and recent evidence suggests that FXR, CAR and PXR are not significantly changed in human placenta during cholestasis [27]. Therefore, whether UDCA can regulate BCRP via activation of a signalling cascade or a modification of maternal hormone levels has yet to be elucidated.

In conclusion, we showed that UDCA administration is associated with a significant upregulation of human placental BCRP supporting a role for BCRP in foetal protection. However, the mechanism underlying this effect remains unknown.
